# Temporal changes in gut microbiota profile in children with acute lymphoblastic leukemia prior to commencement-, during-, and post-cessation of chemotherapy

**DOI:** 10.1186/s12885-020-6654-5

**Published:** 2020-02-24

**Authors:** Ling Ling Chua, Reena Rajasuriar, Yvonne Ai Lian Lim, Yin Ling Woo, P’ng Loke, Hany Ariffin

**Affiliations:** 1 Department of Paediatrics, Faculty of Medicine, University of Malaya, Kuala Lumpur, Malaysia; 2 Department of Obstetrics and Gynaecology, Faculty of Medicine, University of Malaya, Kuala Lumpur, Malaysia; 3 Department of Pharmacy, Faculty of Medicine, University of Malaya, Kuala Lumpur, Malaysia; 4 Centre of Excellence for Research in AIDS (CERiA), University of Malaya, Kuala Lumpur, Malaysia; 5 Department of Parasitology, Faculty of Medicine, University of Malaya, Kuala Lumpur, Malaysia; 6 Department of Microbiology, New York University School of Medicine, New York, NY USA; 7 Department of Paediatrics, University of Malaya Medical Centre, Kuala Lumpur, Malaysia

**Keywords:** Childhood acute lymphoblastic leukemia, Chemotherapy, Microbiome, Microbiota dysbiosis, *Bacteroidetes*, *Bacteroides*

## Abstract

**Background:**

Alteration in gut microbiota has been recently linked with childhood leukemia and the use of chemotherapy. Whether the perturbed microbiota community is restored after disease remission and cessation of cancer treatment has not been evaluated. This study examines the chronological changes of gut microbiota in children with acute lymphoblastic leukemia (ALL) prior to the start-, during-, and following cessation of chemotherapy.

**Methodology:**

We conducted a longitudinal observational study in gut microbiota profile in a group of paediatric patients diagnosed with ALL using 16 s ribosomal RNA sequencing and compared these patients’ microbiota pattern with age and ethnicity-matched healthy children. Temporal changes of gut microbiota in these patients with ALL were also examined at different time-points in relation to chemotherapy.

**Results:**

Prior to commencement of chemotherapy, gut microbiota in children with ALL had larger inter-individual variability compared to healthy controls and was enriched with bacteria belonging to *Bacteroidetes* phylum and *Bacteroides* genus*.* The relative abundance of *Bacteroides* decreased upon commencement of chemotherapy. Restitution of gut microbiota composition to resemble that of healthy controls occurred after cessation of chemotherapy. However, the microbiota composition (beta diversity) remained distinctive and a few bacteria were different in abundance among the patients with ALL compared to controls despite completion of chemotherapy and presumed restoration of normal health.

**Conclusion:**

Our findings in this pilot study is the first to suggest that gut microbiota profile in children with ALL remains marginally different from healthy controls even after cessation of chemotherapy. These persistent microbiota changes may have a role in the long-term wellbeing in childhood cancer survivors but the impact of these changes in subsequent health perturbations in these survivors remain unexplored.

## Introduction

The human gut is colonized by a large number of commensal microorganisms which play important roles in maintenance of good health. Conversely, gut microbiota have also been implicated in the pathogenesis and pathophysiology of certain diseases [1–3]. Despite being a foreign entity to the host and under the constant surveillance of the immune system, gut microbiota are able to coexist synergistically with our immune system [4]. However, perturbations in either gut microbiota or the immune system can potentially affect this mutualistic relationship, and subsequently affect the overall health of an individual [4]. For example, gut microbiota dysbiosis, which is associated with immune activation and inflammation of the intestinal mucosa, is a known key player in the pathogenesis of inflammatory bowel disease [5, 6].

Acute lymphoblastic leukaemia (ALL) is the most common childhood cancer [7]. Advances in treatment strategies have lead to high cure rates [8], but survivors of childhood cancer are at risk of developing many therapy-related late effects, such as metabolic syndrome, cardiovascular disease and musculoskeletal disorders, later in their life [9–11]. In a recent cross-sectional study of young adult survivors of childhood ALL (median age = 26 years old) with a median period of 18.5 years off-chemotherapy, we reported reduced gut microbiota diversity and distinct gut microbiota profile as compared to controls who had no history of cancer. These survivors also exhibited increased markers of immune activation [12] and higher prevalence of metabolic syndrome [13]. However, it is unclear if the microbiota diversity observed in these young adult survivors of ALL is a consequence of chemotherapy exposure during their childhood and has in fact, persisted over time. Understanding this is particularly important in the context of late effects in childhood cancer survivors, which include gastrointestinal complications, chronic inflammation, metabolic syndrome, and cardiovascular disease [9, 10, 13, 14]; conditions, which have all been, associated with gut dysbiosis in the general population [6, 15, 16].

Several recent studies have explored gut microbiota dysbiosis in children diagnosed with ALL [17, 18]. We provide a literature summary of the study designs and findings documented in these studies to give context to our work (Additional file 1: Table S1). Thus far, three studies have highlighted the differences in microbiota profiles between healthy children and those with ALL at diagnosis [17–19]. Children diagnosed with ALL had lower bacteria diversity in their fecal and oral microbiota [17–19]. Fecal microbiota among these children were enriched with certain bacteria including *Bacteroidetes*, *Enterococcaceae* and *Porphyromonadaceae*, while depleted with *Firmicutes*, *Lachnospiraceae* and *Clostridia* [17, 18]. Furthermore, changes in microbiota composition observed during chemotherapy were found to be associated with adverse clinical outcomes [20]. Hakim et al. described that participants with higher baseline relative abundance of *Proteobacteria*, *Enterococcaceae* and *Streptococcaceae* had a greater risk of febrile neutropenia and diarrhea during treatment phase [20]. To date, it is still poorly understood if the host gut microbiota fully recovers in children following remission from ALL.

Gut microbiota dysbiosis has been reported in children diagnosed with ALL but no longitudinal study has thus far tracked the microbiota changes during and after cessation of chemotherapy. Here, we conducted a longitudinal observational study to examine the temporal changes in gut microbiota profile in paediatric patients diagnosed with ALL who underwent chemotherapy and compared these with age- and ethnic-matched controls. Data from this study allowed us to observe the changes in gut microbiota from time of initial cancer diagnosis and the longitudinal impact of chemotherapy on gut microbiota in children with leukemia.

## Methods

### Study participants and sample collection

Seven children diagnosed with ALL at the University of Malaya Medical Centre (UMMC), Malaysia were enrolled. The ALL treatment regimen and risk stratification were according to Ma-Spore ALL 2010 protocol (ClinicalTrials.gov Identifier: NCT02894645) as previously described [21]. The treatment protocol lasts for approximately 2 years and during this period, the patients received trimethoprim-sulfamethoxazole for *Pneumocystis jiroveci* prophylaxis.

Anal swab samples were collected from each patient at three time points: 1) immediately prior to initiation of chemotherapy (sample denoted as pre-chemo), 2) during chemotherapy (sample denoted as during-chemo) and 3) > 3 months after the cessation of all chemotherapy (sample denoted as post-chemo). One anal swab was collected from each healthy control, recruited from children of hospital staff. All controls were free from gastrointestinal conditions, had no antibiotic exposure in 1 month prior to sample collection and were matched to the subjects with ALL by ethnicity, age range and birth mode. We did not exclude ALL patients with recent antibiotic intakes prior to baseline sampling because more than 80% of children with ALL received antibiotics prior to leukemia diagnosis in our medical centre (data not published). This is attributable to the local physician practice of empirical antibiotic treatment for prolonged fever in children; pyrexia of unknown origin being the commonest presenting symptom of ALL in our patients. However, this has also impeded us from including additional group of children with ALL without prior exposure to antibiotics.

### DNA extraction and 16S ribosomal RNA (rRNA) gene sequencing

A total of 39 anal swab samples from seven patients and seven controls were collected and processed as previously described [12]. Briefly, fecal samples were collected using sterile cotton buds and stored at − 80 °C prior to DNA extraction. DNA was extracted from anal swabs using the NucleoSpin® Tissue kit (Macherey-Nagel, Düren, Germany) according to the manufacturer’s protocol. DNA samples were PCR amplified at the hypervariable 4 region of 16S rRNA gene using the protocol modified from Caporaso et al [22] that was previously described [12]. The barcoded amplicons were pooled (multiplexed) at equimolar ratio for the 2x150bp paired-end sequencing using the Illumina MiSeq system (Illumina, San Diego CA, USA).

### 16S rRNA gene sequences processing

Sequencing reads were processed and analysed with QIIME software version 1.8.0 [23] as previously described [12]. The total reads was 2,529,027 with an average reads of 64,847 per sample (standard deviation:±37,277). Performing rarefaction for normalizing variation in sequencing reads across samples has been recommended to reduce the false discovery error due to large variation in sequencing reads across samples [24], even though this may reduce the statistical power to detect rare operational taxonomy units (OTUs) as tested by another group [25]. Hence, we chose to rarefy the 39 samples at the minimal reads in our samples (13,000 reads per sample) prior to downstream analyses. The OTUs were grouped as taxa at different taxonomy classification levels and the relative abundance of each taxon was calculated.

### Analysis of variation in microbiota composition

Alpha diversity for each sample was estimated from OTUs using *alpha_rarefaction.py* workflow implemented in QIIME. Alpha diversity was repeatedly rarefied over 10 depths up to the 13,000 reads depth and bootstrapped 10 times for rarefaction curves analysis. Bray-Curtis dissimilarity was calculated on the OTU compositions using *vegdist* function from “vegan” R package [26] and the resulted matrix was visualized with NMDS plot using “ggplot package”. Statistical differences of the average bacterial community between groups were tested using the *adonis* function from the “vegan” package to perform permutational multivariate analysis of variance (PERMANOVA) with 999 permutations.

### Differential microbial signature community

Differentially abundant bacteria were identified by performing the negative binomial Wald test using the *DESeq* function implemented in “DESeq2” R package [27] which is suggested as a suitable approach for small sample size data [28]. Geometric means of unrarefied OTU abundance counts were calculated and used for normalizing the unevenness in sequencing depth using *estimateSizeFactors* function in ‘DESeq2’ package prior to performing *DESeq* function*.* Due to the nature of high sparsity of microbial data, low abundance OTUs and taxa (that present in less than 10 counts in 30% of the samples) were removed to reduce the number of multiple hypotheses for false discovery rate (FDR) adjustment with Benjamini-Hochberg [29]. Although we collected 1 to 2 samples post chemotherapy per patient, we considered only the last sample from each patient as the post-chemo sample for differential microbial analysis.

### Statistical analysis

Majority of the analysis and graphs were generated using R packages as described above, while certain statistical analysis and graphs were generated using Graphpad Prism (version 7, GraphPad Software, La Jolla, USA). Paired samples were analysed with paired statistical tests while unpaired samples were analysed with unpaired statistical tests. Post-hoc correction was performed for analysis performed on multiple features.

## Result

### Cohort description

Seven ALL patients and seven healthy controls were enrolled, yielding a total of 39 samples. These patients had similar characteristics in terms of age range (2 to 6 years), ethnicity (all Malays), birth-mode (all vaginal delivery) and chemotherapy protocol (Ma-Spore ALL 2010 protocol). Seven healthy children age between 2 to 6 years old were enrolled and their demographic characteristics are homogenous within the group and when compared to the patient group (Table 1). The phase of treatment protocol at which the samples were collected from the ALL patients are indicated in Additional file 1 (Figure S1). One sample was collected upon ALL diagnosis, 1 to 3 samples collected during chemotherapy and 1 to 2 samples collected 3 or more months after chemotherapy cessation. The last sample of each patient was collected between 5 to 9 months post cessation of chemotherapy. In total, each patient with ALL had 4 to 6 samples collected whilst each healthy control contributed a single sample. All patients were exposed to antibiotics within 1 month prior to collection of first sample due to administration of empirical antibiotics for pyrexia of unknown origin, which was the presenting symptom in all patients. Detail regarding clinical features at diagnosis of each patient is documented in Additional file 1 (Table S2).

**Table 1:** Demographic and baseline clinical characteristics of study participants

Subject ID	Group	Ethnicity	Gender	Birth Mode	Gestational Term	^a^ Antibiotics intake prior to diagnosis	^b^ Risk Group	Chemotherapy duration (months)	Follow-up Duration (months)
AL3	Patient	Malay	Female	Vaginal	Term	Yes	Intermediate	27	34
AL4	Patient	Malay	Male	NA	Term	Yes	Standard	25	34
AL8	Patient	Malay	Male	Vaginal	Term	Yes	Standard	25	29
AL10	Patient	Malay	Male	Vaginal	Term	Yes	Intermediate	27	31
AL13	Patient	Malay	Male	Vaginal	Term	Yes	Standard	25	30
AL15	Patient	Malay	Male	Vaginal	Term	Yes	Intermediate	26	31
AL18	Patient	Malay	Male	Vaginal	Post-term	Yes	Standard	24	29
						^a^ Antibiotics intake prior to sampling			
IM18C	Control	Malay	Male	Vaginal	Term	No	–	–	–
ConC1	Control	Malay	Male	Vaginal	Term	No	–	–	–
ConC2	Control	Malay	Male	Vaginal	Term	No	–	–	–
ConC4	Control	Malay	Female	Vaginal	Term	No	–	–	–
ConC5	Control	Malay	Male	Vaginal	Term	No	–	–	–
ConCP3	Control	Malay	Male	Vaginal	Term	No	–	–	–
ConCP5	Control	Malay	Male	Vaginal	Term	No	–	–	–

*NA* no information available

^a^Antibiotic intake within 1 month prior to baseline sample collection

^b^Risk group = ALL patients were assigned to one of the 3 risk groups (ie: standard, intermediate, high), depending on their response to the chemotherapy and special laboratory tests, according to Ma-Spore ALL 2010 treatment protocol

### Distinct microbiota composition in ALL patients which developed some similarities with controls after disease remission

**Fig. 1:**
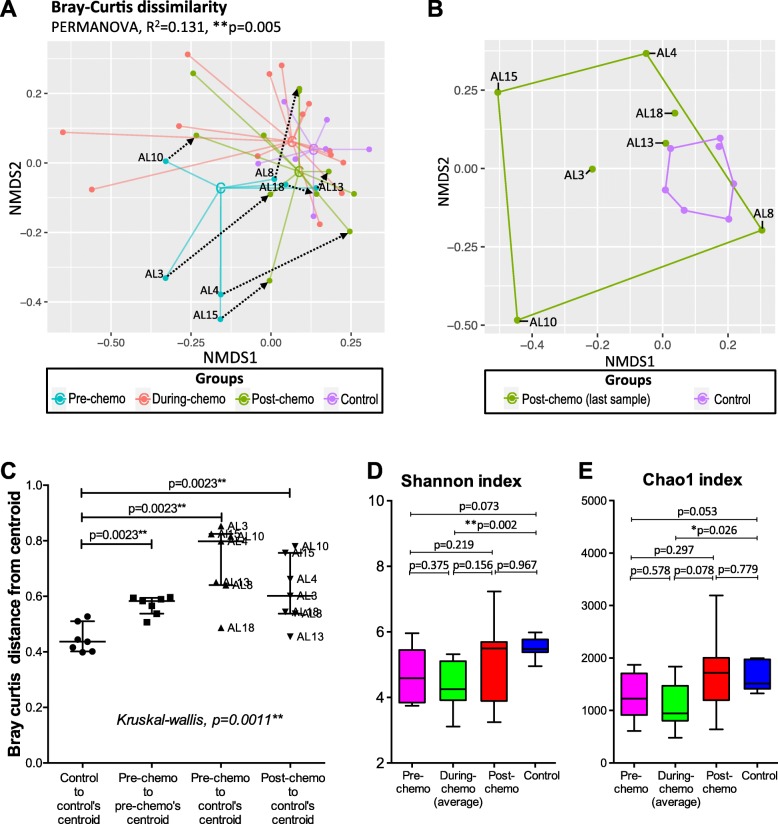
Beta diversity and alpha diversity measures of the ALL patients and controls samples. Bacterial beta diversity was measured with Bray-Curtis dissimilarity distances and visualised on NMDS plot. The samples were coloured according to sampling phase (pre-, during-, or post-chemo) or group (controls) and joined with the respective centroid (labelled with ‘C’). Pre-chemo sample was connected to the last sample Post-chemo with dotted arrow. PERMANOVA shows significant bacterial community differences among the groups (**a**). Bray-Curtis dissimilarity between the post-chemo samples (last timepoint) and controls was also compared (**b**). Microbiota dispersions were assessed based on distances from centroid (**c**). Shannon index and Chao1 index of the pre-chemo, during-chemo (average), post-chemo (last timepoint) samples of ALL patients and controls were also plotted on boxplots and comparison were made with Mann-Whitney tests (for unpaired samples) and Wilcoxon signed-rank test (for paired samples) (**d**, **e**)

Bacterial beta diversity of all the samples was measured with Bray-Curtis dissimilarity distances and ordinated on non-metric multidimensional scaling (NMDS) plot (Fig. 1a). Significant variation in microbiota compositions was observed among the groups (i.e. pre-chemo, during-chemo, post-chemo and control) tested with PERMANOVA (R^2^ = 0.131, *p* = 0.005). The pre-chemo samples were ordinated further from the controls on the NMDS plot, suggesting their microbiota compositions were more different than controls. Microbiota among the pre-chemo samples were also more diverse than that of the controls (Mann-Whitney test, *p* = 0.0023) (Fig. 1c). The pre-chemo samples of four patients (AL3, AL4, AL10 and AL15) had more different microbiota compositions to the other patients and controls (Fig. 1a, c). However, such dysbiotic pattern could be caused by the recent used of antibiotic prior to diagnosis of ALL, which could not be verified without another group of children without ALL but recently treated with antibiotics. During chemotherapy and after cessation of chemotherapy, the patients’ microbiota became more closely resembled that of controls compared to pre-chemo state based on the closer group centroids on the NMDS plot (Fig. 1a). Yet, the post-chemo bacterial composition remained significantly distanced from the control group (Fig. 1b, c).

Microbiota alpha diversity was assessed using Chao1 index and Shannon index (Fig. 1d, e). The pre-chemo samples had slightly lower median alpha diversity than the control samples but were not significantly different (median Shannon index = 4.6 vs 5.5, *p* = 0.073; median Chao1 index = 1226 vs 1512, *p* = 0.053). The average during-chemo samples had significantly lower alpha diversity than control samples (median Shannon index = 4.3 vs 5.5, *p* = 0.002; median Chao1 index = 945 vs 1512, *p* = 0.026). The post-chemo samples and control samples were not different for alpha diversity measures (Fig. 1d, e).

### Differential microbial profile in children diagnosed with ALL and healthy controls

**Fig. 2:**
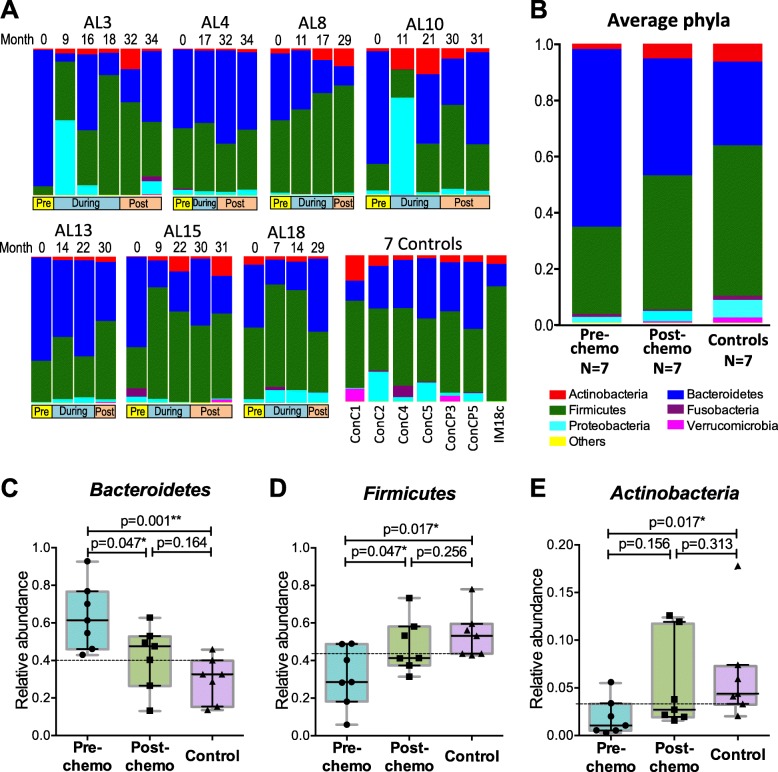
Bacteria phyla compositions in ALL patients and healthy controls. Distribution of the most abundant phyla in each patient across sampling time (labelled as month from baseline) and sampling phase (pre-, during-, and post-chemotherapy), as well as in controls were visualized with stacked barplots (**a**). These top 6 phyla (*Bacteroidetes, Firmicutes, Actinobacteria, Proteobacteria, Fusobacteria* and *Verrucomicrobia*) comprised more than 99% of the abundance, while other taxa were grouped as ‘other’. Average relative abundances of the phyla of the first (pre-chemo) and last (post-chemo) samples of the ALL patients, as well as the controls were also plotted on barplots (**b**). Comparison of the phyla relative abundances between pre-chemo, post-chemo and control groups identified three phyla (*Bacteroidetes*, *Firmicutes* and *Actinobacteria*) that were significantly different among the groups (**c**, **d**, **e**). The lower quartile relative abundance of *Bacteroidetes* and the higher quartile relative abundances of *Firmicutes* and *Actinobacteria* of the control group were indicated with dotted lines

Microbiota of the 39 samples from ALL patients and controls were dominated with bacteria from phyla *Firmicutes*, *Bacteroidetes*, *Proteobacteria* and *Actinobacteria* (Fig. 2a). Pre-chemo samples had a higher average relative abundance of *Bacteroidetes* (64%) than *Firmicutes* (31%). Whereas the post-chemo and control groups had a higher average relative abundance of *Firmicutes* (48% in post-chemo group and 54% in control group) than *Bacteroidetes* (42% in post-chemo group and 30% in control group) (Fig. 2b). Overall, the average phyla distribution in pre-chemo group was different compared to that in controls, while the distribution in post-chemo group demonstrated greater similarities to the control group.

Next, the statistical significance of the differentially abundant phyla between groups was investigated. Samples collected whilst the subjects were undergoing chemotherapy were not included in differential taxa analysis because multiple samples were collected at inconsistent timepoints thus may preclude meaningful analysis. Kruskal-Wallis tests identified three phyla (*Bacteroidetes*, *Firmicutes* and *Actinobacteria*) were significantly different among the pre-chemo, post-chemo and control groups (*p* < 0.05) (Fig. 2c-e). Pre-chemo samples had significantly higher median relative abundance of *Bacteroidetes* than post-chemo samples and control samples (pre-chemo vs controls: *p* = 0.001; pre-chemo vs post-chemo; *p* = 0.047). Of note, all of the pre-chemo samples had higher relative abundance of *Bacteroidetes* than the upper quartile relative abundance of the control group (Fig. 2c). The pre-chemo samples also had a lower median relative abundance of *Firmicutes* and *Actinobacteria* than controls (Fig. 2d, e). No significant difference was identified between the post-chemo and control groups at phylum level.

**Fig. 3:**
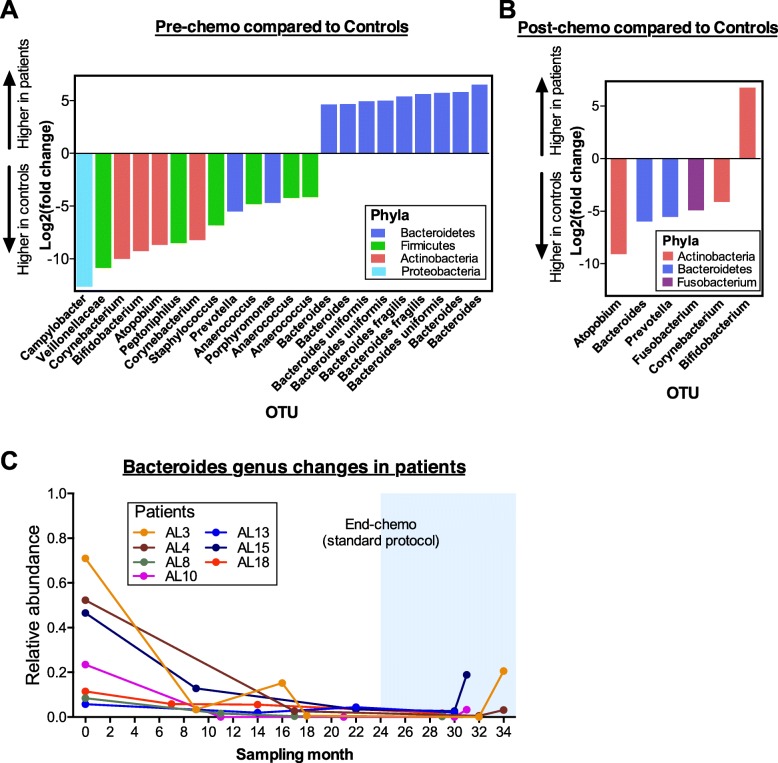
Differentially abundant bacteria were identified between ALL patients and healthy controls. OTU abundances were normalized and compared using Deseq2 analysis pipeline. OTUs with log2 fold change (FC) > 4, base mean > 20 and FDR-adjusted q-values < 0.1 were considered significantly different. Comparison between pre-chemo and control groups identified 13 OTUs that were lower in abundances while 9 OTUs that were higher in abundances in the pre-chemo samples (**a**). Comparison between post-chemo and controls groups identified 5 OTUs that were lower in abundances while one OTU was higher in abundance in the post-chemo samples (**b**). The 9 OTUs that were most abundant among the pre-chemo samples belong to *Bacteroides* genus. Changes in relative abundance of *Bacteroides* genus in each ALL patient were tracked before, during and after cessation of chemotherapy (**c**)

Differentially abundant bacteria OTUs were identified between pre-chemo and post-chemo samples with control samples using DESeq2 analysis (Fig. 3). The pre-chemo samples had 22 OTUs that were significantly different at q-value < 0.1 with log2 fold change > 4 compared to control samples. Thirteen OTUs of which majority belong to *Firmicutes* (6 OTUs) and *Actinobacteria* (4 OTUs) were lower in abundances in the pre-chemo samples compared to controls. Notably, nine OTUs were higher in the pre-chemo samples than controls, of which all these OTUs belonged to *Bacteroides* genus (Fig. 3a). We also noticed that the relative abundance of *Bacteroides* genus reduced during chemotherapy among the ALL patients (Fig. 3c). Comparison between post-chemo and control groups identified six OTUs which were significantly different. Five OTUs had lower abundance in the post-chemo samples, which belong to *Atopobium, Bacteroides, Prevotella*, *Fusobacterium,* and *Corynebacterium* genera, while one OTU belongs to *Bifidobacterium* was present at significantly higher level in the post-chemo samples compared to controls (Fig. 3b). Taxonomy classification, *p*-value, q-value, log2(FC) and base mean of the OTUs that were significantly different in abundance were documented in Additional file 1 (Table S3).

## Discussion

While previous research by other groups have reported gut microbiota dysbiosis in children with ALL before the initiation of chemotherapy [17–19] and during the first year of chemotherapy [18, 20] compared to healthy controls, our study is the first to examine the microbiota changes longitudinally from pre-chemotherapy up to 9 months post completion of chemotherapy. When compared to healthy controls, we observed a larger inter-individual variability and a different bacterial composition among the patients with ALL especially at time of diagnosis (or pre-chemo), consistent with previous studies both in ALL and other types of cancers [17, 18, 30]. Although the microbiota community among the patients with ALL developed greater similarities to the controls (measured with alpha diversity and phyla distribution) post-chemotherapy, there were still differences detected in microbiota composition (measured with Bray Curtis distance) and in abundance of some bacteria OTUs between the groups. Gut microbiota perturbation in our patients during ALL treatment (possibly by chemotherapy and antibiotics) may lead to long-term microbiota dysbiosis. This is not unexpected because previous studies have shown that perturbations in gut microbiota by antibiotics often lead to incomplete microbiota restoration despite cessation of antibiotics [31, 32].

Microbiota at pre-chemo had a lower trend of median alpha diversity than that of the healthy controls. Even though the differences were not significant, the lower trend of alpha diversity in our cohort was in concordance with previous studies measuring both oral and gut microbiota compositions in children with newly-diagnosed ALL [17–19]. We also observed significantly lower bacteria evenness during chemotherapy than in the controls. The same observation has been reported in patients who received conditioning chemotherapy prior to bone marrow transplantation [33, 34] and in mice with chemotherapy-induced mucositis [35]. It is also interesting to note that a previous study showed that gut microbiota diversity in children with ALL decreased during intensive chemotherapy but rebounded during the reduced intensity phase [20]. In our study, majority of the during-chemo microbiota samples were collected during the maintenance (less intensive) phase and thus, we could not verify the above observations [20].

We observed a higher relative abundance of *Bacteroidetes* and lower *Firmicutes* in the patients with ALL at diagnosis compared to healthy children, in concordance with other studies [17, 18]. In particularly, gut bacteria in our patients with ALL were predominantly belonging to *Bacteroidetes* phylum and *Bacteroides* genus. Enrichment of bacteria belonging to the *Bacteroidetes* may be a signature dysbiosis in childhood ALL as this observation is not only found in our study, but has also been reported in three previous studies of children diagnosed with ALL at different study sites [17–19]. A species of *Bacteroidetes,* namely enterotoxigenic *Bacteroides fragilis* has been linked with the pathogenesis of colorectal cancer [36] but its role has never been explored in patient with ALL by previous studies. *B. fragilis* toxin has been shown to be able to induce expression of c-Myc, an oncoprotein, and promote human colonic epithelial cell line proliferation in vitro [37]. Interestingly, we also observed two OTUs affiliated to *B. fragilis* were high in abundance among our patients with ALL at diagnosis. However, we are not able to postulate the role of this bacterium in the leukemogenesis with our study design. Alternatively, the changes observed in pre-chemo samples relative to controls could be associated with antibiotic exposure, as all participants had received a course within a month of sampling. A follow-up study comparing microbiota changes in ALL patients with and without prior antibiotic exposure is needed to confirm the potential influence of bacteria on leukemogenesis.

Our study further extended the sampling timeline to investigate the microbiota composition after cessation of chemotherapy i.e. with the disease in remission. This is important to understand as we previously observed reduced bacteria diversity and microbiota dysbiosis in long-term childhood ALL survivors years after chemotherapy exposure [12]. We found that the median alpha diversity of microbiota in patients with ALL measured five to nine months after completion of chemotherapy was not significantly different from that of the control group. However, while there was no difference in alpha diversity, we detected six OTUs that were differently abundant between the post-chemo patients and controls. Significant differences in both the Bray Curtis dissimilarity distance and OTUs abundances between post-chemo and control samples suggested that the perturbed microbiota in children with ALL did not fully restore to the microbial pattern of the healthy controls despite the potential microbiota modifying factors (including but not limited to chemotherapeutic drugs and antibiotics) have been removed. In this pilot study, we did not find the same differential bacteria that were perturbed in the long-term survivors reported in our previous study [12]. This is not unexpected as subjects in the present study are children while subjects in our earlier study were adults who had ceased chemotherapy more than a decade ago. Additionally, the lifestyle behavior and eating habits of the adult survivors have likely changed considerably. Nevertheless, we do not exclude the possibility that after a longer period of time, the microbiota community in our current cohort of ALL survivors may evolve to acquire a similar dysbiosis pattern as was observed in the long-term survivors.

There are several limitations in our study. Our preliminary findings are based on a small number of subjects and hence may not sensitively identify the bacteria with lower degree of changes. Observations in this study do not suggest causal relationship between microbiota dysbiosis post-chemotherapy with the risk of future health conditions, which would require a longer follow-up study and confirmation study with a bigger group of subjects. Despite previous studies which have shown similar microbiota composition obtained from anal swab and fecal samples [38, 39], we are aware that other studies have on the other hand, demonstrated variation in the gut microbiota composition analyzed by different sampling methods [39, 40]. We performed anal swabs to collect fecal bacteria as opposed to collecting stool samples due to the practicality in the clinic setting and to maintain consistency with our previous study. Moreover, it is challenging to get on-demand fecal samples from young children. In this study, we used 16 s rRNA gene targeted sequencing for microbiota profiling because it is one of the most widely used and robust method to identify and quantify different bacteria taxa within microbial community comprises of large variety of species, but this technique does not allow us to measure the functional genomics of the microbiota [41].

Antibiotics are known to cause alterations in gut microbiota composition [42]. However, studies by Bai et al and Rajagopala et al detected gut microbiota dysbiosis in patients at the diagnosis of ALL regardless of prior exposure to antibiotic treatment [17, 18]. Findings in these studies shown that the lower bacteria diversity in patients with ALL could not be solely explained by the use of antibiotics close to the time of fecal microbiota measurement [17, 18]. Rather, the authors suggested that microbiota dysbiosis in these patients could be influenced not only by antibiotic usage, but also by immune system derangement. We are unable to elucidate the role of malignancy-related altered immunity in causing differences in gut bacterial composition due to the small number of our subjects and the fact that all of them received antibiotics prior to baseline (pre-chemo) sampling. Ideally, we would have enrolled a group of ALL patients not treated with antibiotics. However, it is challenging to enrol children with ALL without prior antibiotic exposure because an audit identified that > 80% of the children diagnosed in our medical centre received empirical antibiotics prescribed at the referring hospitals or by the patient’s primary care physician for fever and presumed infections before the diagnosis of ALL was made. An alternative could have been an additional control group with healthy children (without ALL) treated with antibiotics for fever or other minor illness, which could be included in future studies.

Our main objective was to discover whether gut microbiota pattern in children treated for ALL restored towards a healthy state, as represented by the healthy antibiotic-free children controls, after immune restitution following attainment of disease remission and cessation of chemotherapy. Thus, this study was not designed nor adequately powered to identify the actual causes of gut microbiota perturbation in these children. In addition to antibiotics, gut microbiota dysbiosis in children with ALL can also be affected by other factors such as ethnicity, severity of ALL, treatment intensity, episodes of opportunistic infections, and environment factors, which should be taken into account in future studies.

## Conclusions

In summary, we observed albeit in a small cohort, that gut microbiota dysbiosis was present in children with ALL. Our findings are in concordance with that of the gut microbiota research community particularly regarding enrichment of *Bacteroidetes* among children diagnosed with ALL. We also note that when compared to healthy children, distinctions can be identified in gut microbiota of patients up to 9 months after the cessation of chemotherapy, suggesting that incomplete gut microbiota restoration to resemble the microbial pattern of healthy children. Whether microbiota changes contribute to leukemogenesis in children and/or contribute to the development of inflammation-related late effects in childhood cancer survivors are pertinent questions which remain to be explored.

## Supplementary information

**Additional file 1: Table S1.** Microbiota signature in children diagnosed with and treated for ALL in other studies**. Table S2.** Clinical presentation at ALL diagnosis. **Figure S1.** Sampling timeline for seven ALL patients. **Table S3.** Significantly different OTUs identified using *DESeq2* analysis.

## Data Availability

Additional information included in this study is provided in Additional file 1 comprises of Table S1 (Microbiota signature in children diagnosed with and treated for ALL in other studies), Table S2 (Clinical presentation at ALL diagnosis), Figure S1 (Sampling timeline for seven ALL patients) and Table S3 (Significantly different OTUs identified using *DESeq2* analysis). The 16s rRNA sequencing dataset generated in the current study is archived in the NCBI Sequence Read Archive (SRA) with the accession number PRJNA533024.

## Abbreviations

ALL: Acute lymphoblastic leukemia
FC: Fold change
FDR: False discovery rate
NMDS: non-metric multidimensional scaling
OTU: Operational taxonomy unit
PERMANOVA: permutational multivariate analysis of variance
rRNA: Ribosomal RNA gene
UMMC: University of Malaya Medical Centre
