# Supplementary material for: Temporal changes in gut microbiota profile in children with acute lymphoblastic leukemia prior to commencement-, during-, and post-cessation of chemotherapy
Source: BMC Cancer. 2020 Feb 24;20:151. doi: 10.1186/s12885-020-6654-5 (PMC7041273; doi:10.1186/s12885-020-6654-5)
Supplement: Supplementary file 1 — Additional file 1: Table S1. Microbiota signature in children diagnosed with and treated for ALL in other studies. Table S2. Clinical presentation at ALL diagnosis. Figure S1. Sampling timeline for seven ALL patients. Table S3. Significantly different OTUs identified using DESeq2 analysis. [file 12885_2020_6654_MOESM1_ESM.docx]

### Table S1. Microbiota signature in children diagnosed with and treated for ALL in other studies

**Table S2. Clinical presentation at ALL diagnosis**

| **Subject ID** | **Presenting features at diagnosis** | ***Blast cells (%)** |
| --- | --- | --- |
| **AL3** | Fever 3 days, pallor, anaemia | >20 |
| **AL4** | Fever 1 week, pallor, bruising (skin & arms), knee pain, multiple lymphadenopathy, weight loss (5kg in 2 months), hepatosplenomegaly | 71 |
| **AL8** | Fever 8 weeks, weight loss, joint pain, anaemia | 78 |
| **AL10** | Fever 1 day, respiratory infection 1 week, bruising (lower limbs), pallor, multiple lymphadenopathy, hepatosplenomegaly | 75 |
| **AL13** | Fever 1 week, pallor, jaundice, skin rash, multiple lymphadenopathy | 85 |
| **AL15** | Fever 2 days, pallor, anaemia | 76 |
| **AL18** | Fever 2 weeks, pallor | 90 |
| ***** Blast cells (%) = percentage of blast cells detected in bone marrow aspiration at ALL diagnosis. | | |

**Figure S1. Sampling timeline for seven ALL patients.** Four to six samples were collected from each patient. One anal swab sample was collected at ALL diagnosis, 1-3 samples collected during chemotherapy, 1-2 samples collected 3 months after chemotherapy cessation. Order of samples was indicated with #(number) (eg. #1 denotes first sample). The bar was coloured according the stage of treatment: Pre-, During and Post-chemo. The phase of treatment protocol at which the samples were collected were indicated.

| **Table S3. Significantly different OTUs identified using *DESeq2* analysis** | | | | | | | | | | | |
| --- | --- | --- | --- | --- | --- | --- | --- | --- | --- | --- | --- |
|  | | | | | | | | | | | |
| **Comparison between Pre-chemo samples and healthy control samples** | | | | | | | | | | | |
| **OTU** | **log2**  **(fold change)** | **Base mean** | **p-value** | **q-value** | **Phylum** | **Class** | **Order** | **Family** | **Genus** | **Species** | **Enriched in group** |
| **580629** | 4.63 | 61.2 | 2.99E-03 | 1.68E-02 | Bacteroidetes | Bacteroidia | Bacteroidales | Bacteroidaceae | Bacteroides |  | Pre-chemo |
| **364926** | 4.67 | 64.4 | 9.34E-03 | 3.93E-02 | Bacteroidetes | Bacteroidia | Bacteroidales | Bacteroidaceae | Bacteroides |  | Pre-chemo |
| **197072** | 4.93 | 40.7 | 2.56E-03 | 1.64E-02 | Bacteroidetes | Bacteroidia | Bacteroidales | Bacteroidaceae | Bacteroides | uniformis | Pre-chemo |
| **New.ReferenceOTU350** | 5.00 | 45.5 | 2.92E-04 | 3.01E-03 | Bacteroidetes | Bacteroidia | Bacteroidales | Bacteroidaceae | Bacteroides |  | Pre-chemo |
| **196664** | 5.38 | 232.6 | 2.85E-04 | 3.01E-03 | Bacteroidetes | Bacteroidia | Bacteroidales | Bacteroidaceae | Bacteroides | fragilis | Pre-chemo |
| **193233** | 5.63 | 337.5 | 2.15E-04 | 2.62E-03 | Bacteroidetes | Bacteroidia | Bacteroidales | Bacteroidaceae | Bacteroides | fragilis | Pre-chemo |
| **589071** | 5.73 | 3244.7 | 3.41E-04 | 3.26E-03 | Bacteroidetes | Bacteroidia | Bacteroidales | Bacteroidaceae | Bacteroides | uniformis | Pre-chemo |
| **189920** | 5.80 | 60.0 | 2.73E-05 | 4.07E-04 | Bacteroidetes | Bacteroidia | Bacteroidales | Bacteroidaceae | Bacteroides |  | Pre-chemo |
| **3154070** | 6.50 | 75.6 | 6.42E-05 | 8.60E-04 | Bacteroidetes | Bacteroidia | Bacteroidales | Bacteroidaceae | Bacteroides |  | Pre-chemo |
| **246717** | -12.64 | 1396.8 | 8.53E-13 | 1.14E-10 | Proteobacteria | Epsilonproteobacteria | Campylobacterales | Campylobacteraceae | Campylobacter |  | Control |
| **128382** | -10.86 | 236.2 | 3.88E-08 | 1.73E-06 | Firmicutes | Clostridia | Clostridiales | Veillonellaceae |  |  | Control |
| **984831** | -10.01 | 1105.6 | 2.22E-10 | 1.49E-08 | Actinobacteria | Actinobacteria | Actinomycetales | Corynebacteriaceae | Corynebacterium |  | Control |
| **484304** | -9.26 | 112.8 | 1.07E-06 | 2.87E-05 | Actinobacteria | Actinobacteria | Bifidobacteriales | Bifidobacteriaceae | Bifidobacterium |  | Control |
| **1124877** | -8.69 | 52.1 | 9.68E-07 | 2.87E-05 | Actinobacteria | Coriobacteriia | Coriobacteriales | Coriobacteriaceae | Atopobium |  | Control |
| **1007750** | -8.51 | 67.0 | 5.41E-06 | 1.03E-04 | Firmicutes | Clostridia | Clostridiales | [Tissierellaceae] | Peptoniphilus |  | Control |
| **1050608** | -8.22 | 47.8 | 3.28E-06 | 7.32E-05 | Actinobacteria | Actinobacteria | Actinomycetales | Corynebacteriaceae | Corynebacterium |  | Control |
| **1084865** | -6.82 | 135.9 | 1.12E-05 | 1.87E-04 | Firmicutes | Bacilli | Bacillales | Staphylococcaceae | Staphylococcus |  | Control |
| **851668** | -5.51 | 868.5 | 3.13E-03 | 1.68E-02 | Bacteroidetes | Bacteroidia | Bacteroidales | Prevotellaceae | Prevotella |  | Control |
| **503236** | -4.81 | 32.7 | 2.37E-03 | 1.59E-02 | Firmicutes | Clostridia | Clostridiales | [Tissierellaceae] | Anaerococcus |  | Control |
| **495086** | -4.69 | 831.0 | 2.96E-03 | 1.68E-02 | Bacteroidetes | Bacteroidia | Bacteroidales | Porphyromonadaceae | Porphyromonas |  | Control |
| **930873** | -4.22 | 166.9 | 1.51E-03 | 1.26E-02 | Firmicutes | Clostridia | Clostridiales | [Tissierellaceae] | Anaerococcus |  | Control |
| **495084** | -4.15 | 260.9 | 3.49E-03 | 1.80E-02 | Firmicutes | Clostridia | Clostridiales | [Tissierellaceae] | Anaerococcus |  | Control |
|  | | | | | | | | | | | |
| **Comparison between Post-chemo samples and healthy control samples** | | | | | | | | | | | |
| **OTU** | **log2**  **(fold change)** | **Base mean** | **p-value** | **q-value** | **Phylum** | **Class** | **Order** | **Family** | **Genus** | **Species** | **Enriched in group** |
| **365385** | 6.74 | 47.9 | 8.24E-06 | 4.41E-04 | Actinobacteria | Actinobacteria | Bifidobacteriales | Bifidobacteriaceae | Bifidobacterium |  | Post-chemo |
| **1124877** | -9.08 | 60.0 | 2.55E-07 | 2.73E-05 | Actinobacteria | Coriobacteriia | Coriobacteriales | Coriobacteriaceae | Atopobium |  | Control |
| **355291** | -5.98 | 137.0 | 2.26E-03 | 6.03E-02 | Bacteroidetes | Bacteroidia | Bacteroidales | Bacteroidaceae | Bacteroides |  | Control |
| **851668** | -5.54 | 832.1 | 4.02E-03 | 7.17E-02 | Bacteroidetes | Bacteroidia | Bacteroidales | Prevotellaceae | Prevotella |  | Control |
| **938948** | -4.92 | 246.9 | 8.23E-03 | 9.78E-02 | Fusobacteria | Fusobacteriia | Fusobacteriales | Fusobacteriaceae | Fusobacterium |  | Control |
| **984831** | -4.12 | 752.1 | 3.95E-03 | 7.17E-02 | Actinobacteria | Actinobacteria | Actinomycetales | Corynebacteriaceae | Corynebacterium |  | Control |
